# The role of Co–Ga_2_O_3_ interfaces in methane dry reforming†

**DOI:** 10.1039/d5cy00179j

**Published:** 2025-05-07

**Authors:** Thomas F. Winterstein, Christoph Malleier, Asghar Mohammadi, Roham Talei, Guido Schmitz, Nicolas Bonmassar, Jesus Andrade, Marc Armbrüster, Simon Penner

**Affiliations:** a Institute of Physical Chemistry, University of Innsbruck Innrain 52c A-6020 Innsbruck Austria simon.penner@uibk.ac.at +4351250758003; b Institute for Materials Science, University of Stuttgart Heisenbergstr. 3 70569 Stuttgart Germany; c Materials for Innovative Energy Concepts, Chemnitz University of Technology Straße der Nationen 62 09111 Chemnitz Germany; d Solid State and Material Chemistry, Technical University Darmstadt Peter-Grünberg Str. 12 64287 Darmstadt Germany

## Abstract

As the combination of Co with other non-noble metals is a viable way to improve the catalytic properties of Co in methane dry reforming (DRM), we studied an impregnated Co_3_O_4_/β-Ga_2_O_3_ powder catalyst to understand the influence of Ga and the catalytic role of the Co–Ga_2_O_3_ interface and the intermetallic compound CoGa in DRM. Co_3_O_4_/β-Ga_2_O_3_ undergoes a series of structural transformations during activation by reduction in hydrogen and under DRM conditions. Contact to the CO_2_/CH_4_ mixture without hydrogen pre-reduction yields CoGa_2_O_4_ spinel particles encrusting β-Ga_2_O_3_ without significant DRM activity. Hydrogen reduction transforms Co_3_O_4_/β-Ga_2_O_3_ initially to α-Co/β-Ga_2_O_3_, before it induces reactive metal–support interaction leading to the formation of bimetallic CoGa particles on β-Ga_2_O_3_. Subsequent improved DRM activity can be correlated to the decomposition of the intermetallic compound CoGa: according to *operando* X-ray diffraction CoGa re-transforms into α-Co/β-Ga_2_O_3_ during DRM. Hydrogen pre-reduction is a prerequisite for high DRM activity on Co_3_O_4_/β-Ga_2_O_3_, where intermediarily formed CoGa is decomposed under reaction conditions yielding a pronounced increase in the activity rivalling established noble metal and non-noble metal catalysts. A particular advantage of β-Ga_2_O_3_ is the suppression of coking and Co deactivation, as observed on a Ga-free Co/SiO_2_ catalyst.

## Abstract 2

As the combination of Co with other non-noble metals is a viable way to improve the catalytic properties of Co in methane dry reforming (DRM), we studied Co_3_O_4_/β-Ga_2_O_3_ to understand the influence of Ga and the Co–Ga_2_O_3_ interface in DRM.

## Graphical abstract

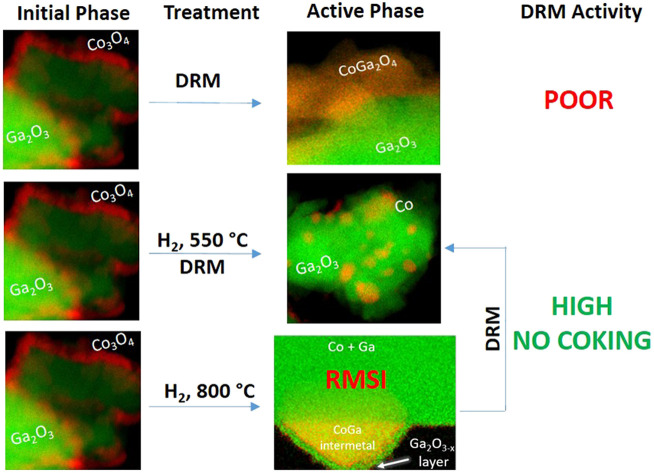

## 1. Introduction

Dry reforming of methane (DRM) is a viable way to transform the two harmful greenhouse gases carbon dioxide (CO_2_) and methane (CH_4_) into useful syngas (*i.e.*, a hydrogen (H_2_) – carbon monoxide (CO) mixture). DRM is a strongly endothermic reaction and, thus, requires rather high reaction temperatures of 600–1000 °C.^1–3^ The main DRM reaction (eqn (1))

1 CO_2_ + CH_4_ ↔ 2H_2_ + 2CO

thereby is only part of a complex reaction network that also comprises reactions relevant for catalyst regeneration (*e.g.*, the reverse Boudouard reaction), reactant activation (*e.g.*, methane or carbon dioxide decomposition) or loss of selectivity (*e.g.*, the reverse water–gas shift reaction). The latter is important on oxides and oxide-related materials, which through surface-bound or oxygen vacancy-bound mechanisms can beneficially or detrimentally contribute to the overall catalyst activity and selectivity.

Material-wise, non-noble metal catalysts based on Ni or Co have evolved as alternatives to excellently performing, yet expensive noble metal-based catalysts.^1,3–6^ Whereas Ni is favored due to its outstanding catalytic DRM performance, large-scale application is hindered by sintering and coking, which diminishes the activity quickly. While slightly less active than Ni, Co offers better anti-coking properties and higher sintering stability.^7–9^ Thus, Co-based materials are considered as one of the most promising future DRM materials.^4^ Several approaches were followed in the past decade to improve the catalytic properties of Co-based catalysts, especially with respect to enhancing their oxidation resistance.^4^ The addition of a second metal (*e.g.*, Ni, Ce, Fe or noble metals) can significantly improve the binding strength of adsorbed oxygen species.^10–15^ Specifically, co-alloying with Ni or Fe was shown to improve the dispersion of metal species and to accordingly enhance the sintering resistance, increase the amount of active sites for carbon dioxide or methane activation and beneficially influence the redox properties.^16–20^ As such, Co-based catalysts have been developed rapidly to improve their catalytic and physico-chemical characteristics.

As a viable strategy to improve the catalytic performance of supported Co-based catalysts in DRM, exploitation of an enhanced metal–support interaction strength has been pointed out. Derived from general principles of metal–support interaction processes,^21^ upon reduction such an interaction may manifest itself in three possible ways: (i) strong metal–support interaction, *i.e.*, coating of active Co metal by a partially reduced support, (ii) electronic metal–support interaction, *i.e.*, electron transfer between Co metal and support and (iii) reactive metal–support interaction leading to the formation of Co-based alloys or intermetallic compounds by reaction of the supported Co nanoparticles with the fully reduced support.^21^ Regarding the latter, research has been particularly devoted to bimetallic Co-based materials.^4^ Generally, alloys and intermetallic compounds are one of the most rapidly evolving materials classes with prime importance in a variety of heterogeneously catalyzed reactions.^22–25^

A topic that is connected to reactive metal–support interaction is the reversibility of alloy and intermetallic compound formation and their potential structural stability.^26^ Those compounds, also if formed by reduction of a supported metal catalyst (*i.e.*, oxide-supported alloy or intermetallic compound particles), can be structurally unstable depending on the experimental conditions. Preferential leaching of one component may lead to decomposition and formation of an intermetallic compound – oxide (partial decomposition) or a metal–oxide interface (full decomposition).^26^ In either case, the alloy or intermetallic compound particles act as a catalyst precursor, which is activated upon decomposition. Although at first glance detrimental, this process can be useful, if carried out in a controlled way. Closely connected to the process of exsolution of metal particles from a host lattice,^27^ steered decomposition might lead to extended metal–oxide interfaces with controlled particle sizes, better-distributed or better-anchored particles with improved sinter stability or coking properties.^26^ This decomposition of intermetallic compounds and particles has been frequently observed in a variety of reactions,^26^ including methanol steam reforming (*e.g.*, Cu_51_Zr_14_,^28–30^ ZnPd,^31–35^ GaPd_2_,^36–40^ InPd,^40–42^ or Cu_2_In (ref. 43)) or methane dry reforming (*e.g.*, Ni_5_Zr,^44–46^ or Pd_2_Zr (ref. 46–48)). The common denominator is the delicate balance between high (no or only partial decomposition) and low stability (full decomposition). This was scrutinized, *e.g.*, for Cu/In_2_O_3_,^43^ Pd/In_2_O_3_ (ref. 49) and Pt/In_2_O_3_ (ref. 50) previously. Catalytic-wise, such a decomposition often invokes a so-called bifunctional synergism with shared catalytic duties of reactant activation. With respect to DRM, methane activation usually proceeds on the metal surface, carbon dioxide is accordingly adsorbed and activated on acidic or basic oxide support sites.^1–3^ A delicate balance of the latter is important, as too strong acidic sites facilitate carbon formation through easier methane decomposition and too strong basic sites promote the Boudouard reaction and the oxidation of active metal.^4^

We have, thus, selected Co as the lead-element, as its DRM activity rivals that of Ni and the deactivation of Co-based materials in DRM is essentially connected to the strongly size-dependent balance between coking and cobalt oxidation.^4,51^ Selection of the second component is driven by the eventual co-operative interaction between the two metals and by the possibility to enter a state of reactive metal–support interaction. For Ga-based compounds, this has been already shown for Pd/Ga_2_O_3_.^38,39^ In due course, as Ga has a significantly higher oxidation tendency compared to Co,^52^ we expect an eventually formed Co–Ga intermetallic compound^53,54^ to at least partially decompose under DRM conditions into Co and Ga_2_O_3_. To assess the complex structural dynamics of eventual reactive metal–support interaction and to study the reversibility of Co–Ga intermetallic compound formation, we use a conventionally wet-impregnated Co_3_O_4_/β-Ga_2_O_3_ powder material. β-Ga_2_O_3_ as an amphoteric oxide with neither too strong acidic nor basic sites^55^ is expected to yield an optimum surface-chemical compromise to activate carbon dioxide and to facilitate the dehydrogenation of methane, thus providing high DRM activity.^56^ In addition, recent experiments on a Rh/Ga_2_O_3_ photothermal catalyst for DRM applications pointed out the important role of Ga_2_O_3_ defects for CO_2_ activation.^57^ The results are expected to eventually pave the way to accordingly assess the redox activation and DRM properties of single-phase Co–Ga intermetallic compounds, as pointed out for, *e.g.*, the Cu–In system already.^43^ Co_3_O_4_/β-Ga_2_O_3_ is subjected to *operando* and *in situ* structural characterization by powder X-ray diffraction (PXRD), atomically resolved (scanning) transmission electron microscopy (STEM) – EDX (energy-dispersive X-ray spectroscopy) measurements and X-ray photoelectron spectroscopy, and accordingly correlated to DRM activity for establishing reliable structure–activity correlations.

## 2. Methods and materials

### 2.1. Synthesis of materials

The Co_3_O_4_/β-Ga_2_O_3_ sample was prepared by wet impregnation. 1.8 g β-Ga_2_O_3_ were added to a Co(NO_3_)_2_·6H_2_O solution (referenced to 0.003394 mol Co dissolved in 150 mL water, yielding a Co concentration of 2.3 mol L^−1^) under constant vigorous stirring to obtain a nominal content of 10 wt% Co_3_O_4_. Subsequently, the water was slowly evaporated by heating to 60 °C at a rate of 2 K min^−1^. The remaining powder was collected, ground and calcined at 600 °C for 2 h in air (heating rate: 10 K min^−1^). PXRD patterns (*cf.*Fig. 2) indicate the sole presence of the Co_3_O_4_/β-Ga_2_O_3_ starting material. Co/SiO_2_ (nominal content 10 wt% Co_3_O_4_) was prepared the same way using Aerosil 380 powder (Degussa) and the same nominal Co_3_O_4_ loading.

Bulk intermetallic CoGa was used as reference for the XPS measurements and 1 g was obtained by high-temperature synthesis. Stoichiometric amounts of Co (pieces, ChemPur 99.9%) and Ga (pellets, ChemPur, 99.999%) were mixed in a glassy carbon crucible and subjected to high-frequency heating (TruHeat HF 5005, TRUMPF Hüttinger) in inert argon atmosphere to 1250 °C. After 3 minutes, the sample was allowed to cool to ambient temperature. Characterisation by XRD resulted in the aimed-for single-phase CoGa sample with the CsCl-type of crystal structure.^58^

### 2.2. Structural characterization

*Ex situ* structural analysis of benchmark materials after selected treatments was carried out using a Rigaku SmartLab-SE instrument in Bragg–Brentano geometry (Co-Kα, *λ* = 1.7890 Å) using a D/teX Ultra 250 compound silicon strip 1D-detector (Rigaku, Tokyo, Japan). The ground sample was placed on a glass-sample holder and the patterns were recorded in a range of 2*θ* 5° to 90° with a step width of 0.01°. The program TOPAS 5.0 by Bruker was utilized to analyze the X-ray diffraction patterns using Rietveld refinement with a full axial model. A double-Voigt approach was applied to calculate the crystallite sizes.

*In situ* and *operando* X-ray diffraction experiments on Co_3_O_4_/β-Ga_2_O_3_ were performed using a Rigaku ReactorX on a Rigaku SmartLab XE 3 kV in Bragg–Brentano geometry (Co Kα radiation, *λ* = 1.17890 Å, D/teX Ultra 250 1D-detector). The reactor chamber was flushed with He with a flow rate of 40 mL min^−1^ for one hour before starting the experiments. These consisted of two consecutive steps. The first step was a reduction with hydrogen at 800 °C, using a gas mixture of H_2_/He (10% vol. H_2_) with a flow rate of 40 mL min^−1^. Before starting the reduction, an XRD scan from 2*θ* 5° to 100.5° was conducted. The mixture of H_2_/He was subsequently admitted to the system 15 min before starting to heat the sample with a temperature ramp of 25 K min^−1^ to 800 °C. Every minute, continuous XRD scans in the range of 2*θ* 48° to 52° were performed. At 800 °C, a full XRD scan was performed from 2*θ* 5° to 100.5°. The system was then cooled down to 50 °C with a temperature ramp of 25 K min^−1^, flushed with He with a flow rate of 40 mL min^−1^ before another full XRD scan was recorded. Subsequently, the DRM step was conducted, starting from 50 °C to at 800 °C (heating rate 5 K min^−1^) using a 1 : 1 : 1 gas mixture of CO_2_, CH_4_ and He with a flow rate of 20 mL min^−1^ each. During heating, every minute continuous XRD scans were performed from 2*θ* 48° to 52° and a full scan was performed at 800 °C. A final full XRD scan was taken after the system was cooled down to ambient temperature with 25 K min^−1^ and flushing with He. A mass spectrometer (GSD 350 Omnistar, Pfeiffer Vacuum) was used to follow the changes in the gas mixtures, as well to track products during the reduction and DRM steps. CO_2_, CO, H_2_O, CH_4_, He and H_2_ were assigned to *m*/*z* 44, 28, 18/17, 16, 4 and 2, respectively. The data were normalized using He as internal reference and the signals were smoothed with the Savitzky–Golay filter (20 point window and second polynomial order).

### 2.3. Catalytic testing

200 mg of sample powder was placed in a bed of silica wool inside a 7 mm (inner diameter) silica tube flow reactor for both H_2_ pre-treatment and catalytic measurements. A Linn HighTerm FRH 25/150/1100 furnace was used for all heating cycles. H_2_ treatments consisted of a continuous isothermal flow of 20 mL min^−1^ H_2_ at 800 °C for 10 min after heating the sample in the H_2_ stream from 25 °C to 800 °C with a rate of 20 K min^−1^. After the hydrogen pre-treatments, the samples were re-cooled in hydrogen to 25 °C before switching to the DRM mixture. For catalytic measurements CH_4_, CO_2_ and He were mixed 1 : 1 : 1 with a flow rate of 20 mL min^−1^ each and a heating rate of 10 K min^−1^ from 25 °C to 800 °C, followed by an isothermal period at 800 °C for 30 min. An external S-type thermocouple was placed in close contact to the reactor tube to ensure the correct temperature reading. The output gas was directly detected by an on-line quadrupole mass spectrometer (Balzers QME 125). CO_2_, CH_4_, CO, H_2_ and He were measured at their respective *m*/*z* ratios of *m*/*z* 44, 28, 16, 4 and 2. The He signal was used to normalize the gas mixture. Relevant fragmentation patterns have been considered in the analysis. To minimize condensation from potential H_2_O formation, silica gel was placed at the exhaust of the furnace. For the display of the catalytic data, we show the conversion of the relevant signals as a qualitative measure of the catalytic activity, as due to the ongoing structural transformations during the DRM reaction, active-site-normalized reaction rates and consequently, TOF values, cannot be reliably calculated. The conversion for each gas phase is calculated as a fraction of the mass spectrometer (MS) ion current [E^−10^ A] divided by the MS signal at room temperature after calibrating the flow rate of each gas phase. The measured He signal was used to correct fluctuations in the MS signals. For CO_2_ the calculation is as follows:
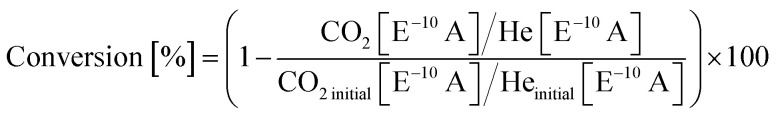
Since the CO_2_ conversion is indicative of CO, and CH_4_ conversion of H_2_ production, the H_2_/CO ratio was calculated from these conversions, respectively.

### 2.4. (Scanning) transmission electron microscopy

We performed STEM measurements on a probe-corrected FEI Spectra300. All specimens were prepared by the drop-casting method, where a suspension consisting of isopropanol and to-be-investigated particles is dropped on a lacey carbon grid. We used high-angle annular dark field (HAADF) imaging in combination with energy-dispersive X-ray (EDX) spectroscopy (specimen-tilt independent Super-X EDS-system) for the characterization of the morphology and the chemical composition of the catalysts. For the quantitative EDXS results we used the Schreiber–Wims ionization cross-section and a parabolic background model, which are implemented into the Velox software.

Scanning electron microscopy (SEM) and corresponding energy-dispersive X-ray spectroscopy measurements were performed on a field-free analytical TESCAN Clara ultra-high-resolution scanning electron microscope operated at 10 kV. EDX maps were collected using an Oxford Ultim Max 65 mm^2^ detector.

### 2.5. X-ray photoelectron spectroscopy

Chemical analysis of the near-surface region was carried out using a Thermo Fisher X-ray spectrometer with Al-K_α_ radiation. For all measurements, samples were fixed on stainless steel holders using conductive carbon tape. Qualitative analysis was based on the Ga 2p, Co 2p, O 1s and C 1s high-resolution spectra. Chemical shifts were internally calibrated to the O 1s component at 531.8 eV. Fitting of the spectra by different Co 2p components and oxidation states was performed using literature-reported constraints for the full-width-at-half maximum and the binding energies, where applicable.^59^ Background correction has been done using Tougaard-type functions.

## 3. Results and discussion

### 3.1. Dry reforming performance of Co_3_O_4_/β-Ga_2_O_3_

**Fig. 1:**
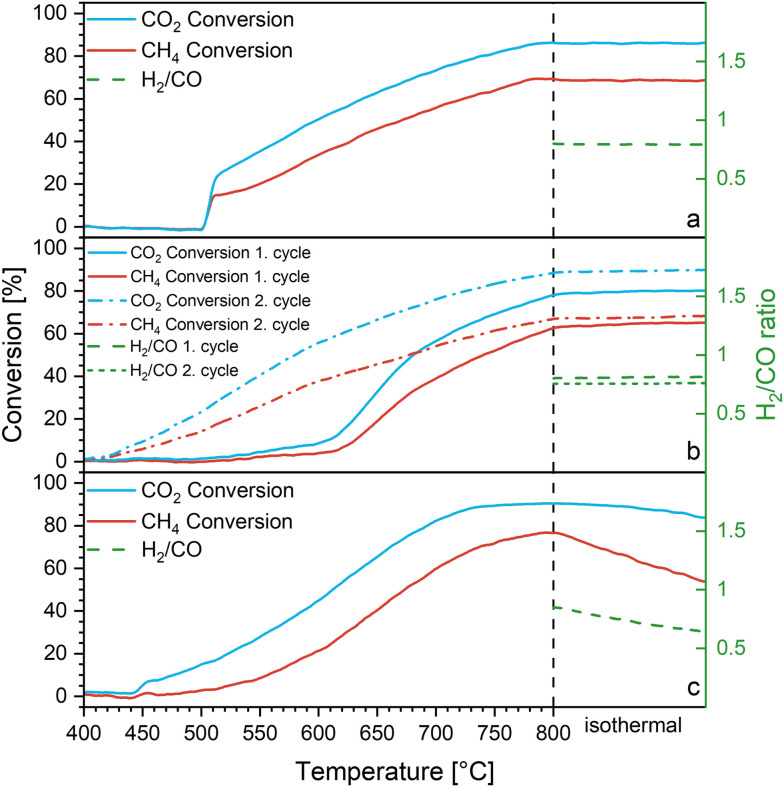
Methane dry reforming profiles of Co_3_O_4_/β-Ga_2_O_3_ during DRM operation after hydrogen reduction at 550 °C for 1 h (panel a) and after prior hydrogen reduction at 800 °C for 10 min (panel b). Panel c shows the DRM performance of Co/SiO_2_ after initial hydrogen reduction at 450 °C for 10 min. Sample mass: 200 mg each, GHSV: 18 L g^−1^ h^−1^, heating rate: 10 K min^−1^. The respective H_2_/CO ratios are highlighted on the right axis.

**Fig. 2:**
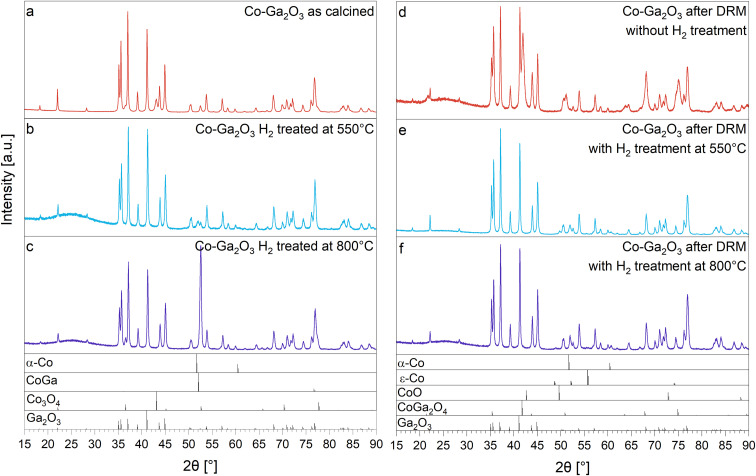
XRD patterns of Co_3_O_4_/β-Ga_2_O_3_ in panel a: as-calcined state, panel b: after hydrogen reduction treatment at 550 °C, panel c: after hydrogen reduction treatment at 800 °C and panel d: after DRM operation at 800 °C without prior hydrogen, panel e: after DRM operation at 800 °C with prior hydrogen reduction at 800 °C, panel f: after DRM operation at 800 °C with prior hydrogen reduction at 550 °C. Structure ICSD-numbers and references: α-Co 136039,^63^ ε-Co 76633,^64^ CoO 9865,^65^ CoGa_2_O_4_ 172183,^66^ CoGa 657494,^67^ β-Ga_2_O_3_ 3423423,^68^ Co_3_O_4_ 36256.^69^

We have performed methane dry reforming experiments on two different samples: Co_3_O_4_/β-Ga_2_O_3_ with and without hydrogen pre-reduction and referenced the catalytic activity to a corresponding Ga-free Co_3_O_4_/SiO_2_ catalyst with the same nominal Co_3_O_4_ loading (Fig. 1). Without hydrogen pre-reduction (Fig. S1†), the sample is hardly active: limited catalytic activity is observed at around 700 °C, and conversions of CO_2_ and CH_4_ of only 10% and 5% are reached after the 30 min isothermal section at 800 °C. The deviation of the CO_2_ and CH_4_ profiles already indicate a competing reverse water–gas shift activity, most likely mediated by the β-Ga_2_O_3_ support. Reference DRM profiles of β-Ga_2_O_3_, SiO_2_, the blank reactor filled with quartz wool and Co_3_O_4_ are provided in Fig. S2.† No activity is observed for the first three. Panel d in Fig. S2† (pure Co_3_O_4_) reveals that the situation is more complex: as anticipated, Co_3_O_4_ itself is DRM active starting at around 740 °C. CO_2_ and CH_4_ conversions of *ca.* 50% and 40% are observed. Beyond 760 °C, the catalyst strongly deactivates by coking. If supported on β-Ga_2_O_3_, the activity of Co_3_O_4_ is also affected by the ongoing transformation into the DRM-inactive CoGa_2_O_4_ spinel phase (see Fig. S2e†). Synthesis temperatures for this spinel have been reported as low as 700 °C,^60^ so the active DRM window directly coincides with the formation temperature of the spinel phase. Previous work has shown, that β-Ga_2_O_3_ is an efficient water–gas shift catalyst above 600 °C, capable of catalyzing this reaction *via* two separate reaction mechanisms: a formate-bound one, and a more vacancy-dominated pathway.^61^ Hydrogen reduction prior to DRM operation (Fig. 1a and b) changes the scenario drastically. We have performed hydrogen reduction at two different maximum temperatures: 1 h at 550 °C and 10 min at 800 °C, respectively. As will be discussed in the context of Fig. 2, the difference between the two pre-treatments is that reduction at 550 °C causes only reduction of Co_3_O_4_ to Co metal, whereas the latter induces the formation of a CoGa intermetallic compound before DRM operation. Starting with the DRM activity in the first DRM cycle after hydrogen pre-reduction at 800 °C (Fig. 1b), after a period of slow activation between 500 °C and 650 °C, the material becomes very active. CO_2_ and CH_4_ conversion levels of 80% and 70% are reached in the isothermal period at 800 °C, respectively. The considerably higher CO_2_ conversion is once again notable, getting increasingly more pronounced as the reaction temperature increases. This again points to the selectivity-spoiling reverse water–gas shift reactivity of β-Ga_2_O_3_.^61^ Put into literature perspective, within the framework of slightly different experimental conditions, the Co_3_O_4_/β-Ga_2_O_3_ catalyst rivals similar Co-based catalysts modified with either noble- or non-noble metals. Specifically, Co_3_O_4_/β-Ga_2_O_3_ after hydrogen reduction consistently outperforms Ru-Co catalysts supported on TiO_2_ or α-Al_2_O_3_ (for the former, CO_2_ and CH_4_ conversions of 75% and 55% were reported at 850 °C respectively; for the latter CO_2_ and CH_4_ conversions of 35% and 29% were reported at 900 °C (ref. 4)) and equals those supported on mesoporous SiO_2_ (CH_4_ conversion 74% (ref. 4)). In due course, the performance of Co_3_O_4_/β-Ga_2_O_3_ matches or exceeds Fe-doped Co alloy particles supported on activated carbon materials or those exsolved from mixed or layered perovskite materials like GdCo_0.5_Fe_0.5_O_3_ (CH_4_ and CO_2_ conversions both below 30% at 800 °C (ref. 62)) or PrBaFeCoO_5+*δ*_ (CH_4_ and CO_2_ conversion of <20% and 36% at 900 °C, respectively^4^).

As will be discussed in the context of the *operando* XRD experiments in Fig. 4, the CoGa intermetallic compound is stable up to *ca.* 550 °C, before it decomposes into metallic Co and additional β-Ga_2_O_3_. The strong acceleration of catalytic activity at around 600 °C in the DRM profiles of Fig. 1b (first cycle) is, therefore, associated with the transition from CoGa/β-Ga_2_O_3_ to Co/β-Ga_2_O_3_.

**Fig. 3:**
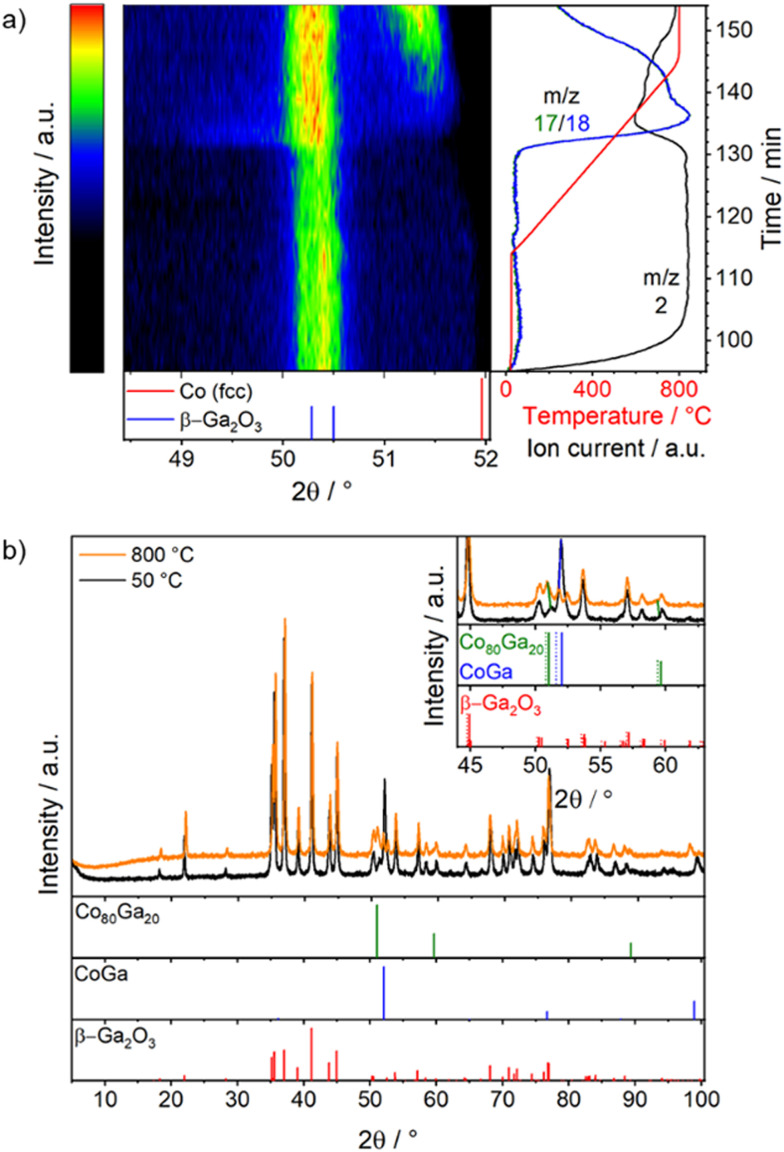
*In situ* XRD patterns collected on Co_3_O_4_/β-Ga_2_O_3_ during reduction in hydrogen: panel a: patterns collected during the reduction step with associated mass spectra of *m*/*z* 18, 17 and 2; panel b: XRD patterns at 800 °C during reduction and after the reduction step on the sample cooled to 50 °C. Gas mixture: H_2_/He (10% vol. H_2_) with a flow rate of 40 mL min^−1^. Heating rate: 25 K min^−1^. The dotted lines in the inset are the calculated XRD patterns of the modified lattice parameters due to thermal expansion. Structure references: Co (fcc)^70^ β-Ga_2_O_3_,^71^ CoGa,^72^ Co80Ga20.^73^

**Fig. 4:**
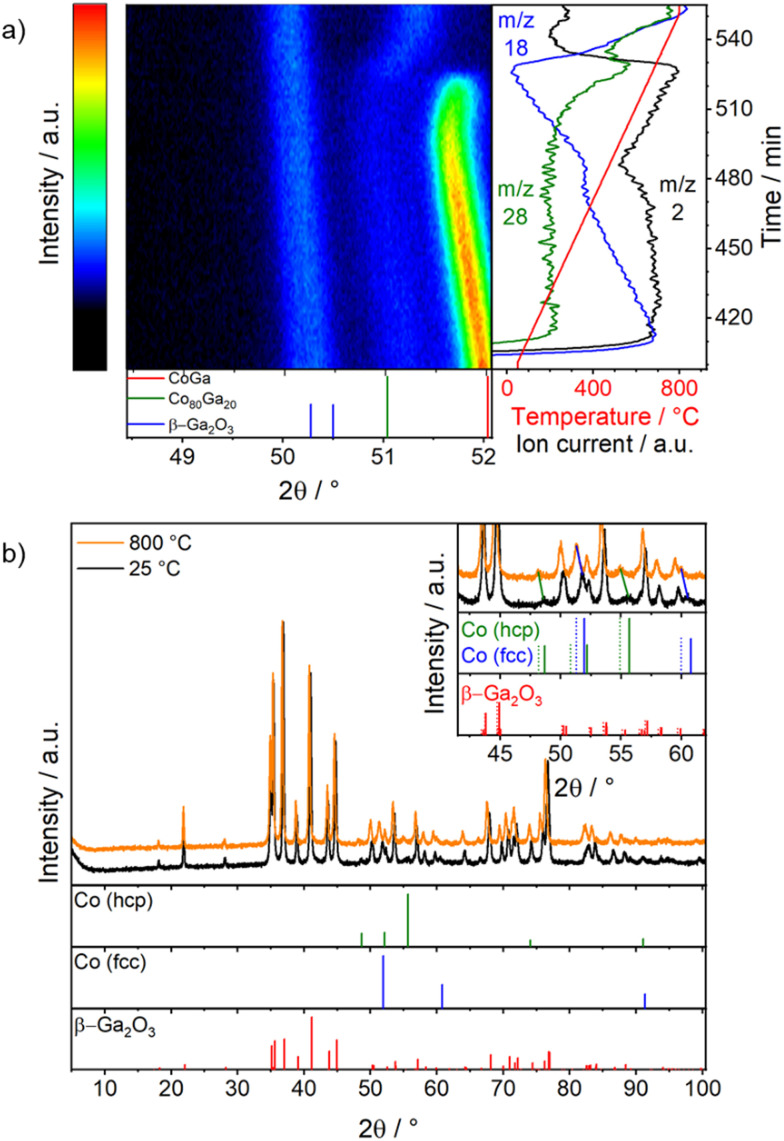
*Operando* XRD patterns collected on Co_3_O_4_/β-Ga_2_O_3_ during DRM after the hydrogen reduction step shown in Fig. 5: panel a: patterns collected during DRM with the associated mass spectra of *m*/*z* 28, 18 and 2; panel b: XRD patterns at 800 °C during DRM and after DRM operation and cooling the sample to room temperature. Gas mixture: 1 : 1 : 1 ratio of CO_2_, CH_4_ and He with a flow rate of 20 mL min^−1^ each. Heating rate: 5 K min^−1^. The dotted lines in the inset are the calculated XRD patterns of the modified lattice parameters due to thermal expansion. Structure references: Co (fcc),^70^ Co (hcp),^74^ β-Ga_2_O_3_,^71^ CoGa,^72^ Co80Ga20.^73^

After pre-reduction at 550 °C, the respective DRM profile (Fig. 1a) exhibits a much lower onset temperature of 520 °C and conversion levels of both CO_2_ and CH_4_ are much higher at comparable temperatures. According to the structural characterization of the benchmark states (Fig. 2) and the TEM analysis and the *in situ* and *operando* stability tests (*cf.*Fig. 3, 4, 6 and 7), DRM after pre-reduction at 550 °C probes metallic Co on β-Ga_2_O_3_, whereas DRM after pre-reduction at 800 °C reveals the activity of the CoGa compound during DRM operation up to *ca.* 550 °C. At higher reaction temperatures – as after pre-reduction at 550 °C – Co/β-Ga_2_O_3_ is probed (we have accordingly assessed the Co crystallite sizes after the respective hydrogen treatments at 550 °C/800 °C and subsequent DRM runs to be 28 nm and 35 nm, respectively. The respective SEM data indicate comparable Co particle sizes of around 50–100 nm after both treatments, excluding substantial Co crystallite and particle size effects). To test this hypothesis, we have subjected the 800 °C pre-reduced sample to a second consecutive DRM cycle (*i.e.*, in the Co/β-Ga_2_O_3_ state after decomposition of CoGa in the first DRM run). As anticipated, the respective DRM profile (dashed lines in Fig. 1b) matches not only that after a pre-reduction at 550 °C, but also that of a Ga-free Co_3_O_4_/SiO_2_ reference catalyst after pre-reduction (Fig. 1c, hydrogen pre-reduction at 450 °C for 10 min). The latter reaches final conversion levels of 84% (CO_2_) and 76% (CH_4_) at 800 °C, respectively. A closer look at the DRM profiles indicates a two-fold beneficial role of β-Ga_2_O_3_: it lowers the initial onset temperature by about 50 °C (400 °C *vs.* 450 °C on Co_3_O_4_/SiO_2_) and it suppresses catalyst deactivation in the isothermal section at 800 °C at least in the studied time-on-stream regime. While Co_3_O_4_/SiO_2_ shows clear signs of deactivation after the pre-reduction-DRM cycle, the latter is virtually absent for Co_3_O_4_/β-Ga_2_O_3_, irrespective of the nature of the hydrogen pre-treatment. As will be discussed in the context of Fig. S3 and S4,† this is essentially due to the different coking behavior. On the basis of several SEM images after a hydrogen treatment at 800 °C and a subsequent DRM run, Co particles sizes between 50 nm and 150 nm have been determined on Co_3_O_4_/SiO_2_ and Co_3_O_4_/β-Ga_2_O_3_, ruling out substantial particle size or Co dispersion-related effects on DRM activity or coking resilience.

### 3.2. Structural characterization of selected benchmark states after reduction and DRM operation

To identify benchmark phases during hydrogen reduction and DRM operation, Fig. 2 shows that after initial calcination (panel a), Co_3_O_4_ (space group: *Fd*3̄*m*)^69^ on β-Ga_2_O_3_ (space group: *C*2/*m*^68^) is present. The diffractograms after reduction and DRM already indicate the high propensity of Co_3_O_4_/β-Ga_2_O_3_ to undergo structural transformations. According to XRD (panel d), during DRM operation without prereduction Co_3_O_4_/β-Ga_2_O_3_ transforms into CoGa_2_O_4_/Ga_2_O_3_. CoGa_2_O_4_ is a spinel-type bimetallic oxide (space group *Fd*3̄*m*)^75^ with reported dominant oxygen vacancy chemistry, enabling rich electrochemical applications.^60,66,76^ As shown in Fig. S1,† this structural transformation is not associated with significant DRM activity. After hydrogen reduction at 550 °C, Co_3_O_4_ is reduced to metallic cubic Co (panel b). Hydrogen reduction at 800 °C, however, allows to enter a state of reactive metal–support interaction (panel c, RMSI)^21^*via* formation of the intermetallic compound CoGa (space group: *Pm*3̄*m*^67^) on β-Ga_2_O_3_ (an XRD pattern of the synthesized, untreated CoGa intermetallic compound is provided in Fig. S5†). The latter term is used to classify reactions between supported elemental nanoparticles and the support, usually leading to irreversible oxide reduction and formation of intermetallic compound particles on the supporting oxide surface. This type of reactive metal–support interaction is a frequently observed phenomenon in catalysis and concerns both noble- and non-noble metal containing catalysts. Recently, the term has also been introduced to account for according changes under non-reducing conditions.^77^ Although initially restricted to easy reducible oxides, the term is also used to describe the associated changes on hard-to-reduce oxides like β-Ga_2_O_3_. Specifically, Pt, Pd, Rh and Ni particles have been all found to enter such a state if supported on β-Ga_2_O_3_, resulting in GaPt_2_, GaPd_2_, GaPd, Ga_3_Rh_5_ and GaNi_3_. For many of those systems, distinct structure–activity correlations have been established.^21^

Panels e and f reveal that upon subsequent DRM operation metallic Co is stable (referring to hydrogen pre-reduction at 550 °C, panel e) and that CoGa is unstable and decomposes into cubic α-Co/β-Ga_2_O_3_ with trace amounts of hexagonal ε-Co present after cooling (panel f). The details of the Rietveld refinement are summarized in Table S1.†

The respective PXRD patterns for the Co/SiO_2_ catalyst (Fig. S3†) – apart from the consistent presence of metallic Co and SiO_2_ in the as-calcined and hydrogen pre-reduced/DRM state – indicate the formation of graphitic carbon species after DRM operation. Connecting to the catalytic results in Fig. 1, this explains the deactivation feature in the isothermal section for Co/SiO_2_ – in striking contrast to Co_3_O_4_/β-Ga_2_O_3_.

As the PXRD patterns after DRM operation already indicate a different coking behavior and revealed (partially) crystalline graphite-like carbon species on Co_3_O_4_/SiO_2_ after DRM operation in contrast to Co_3_O_4_/β-Ga_2_O_3_, SEM analysis was carried out to pinpoint the exact location of these carbon species (Fig. S4†). After hydrogen pre-reduction at 550 °C (Fig. S4b,† in comparison to the as-calcined state shown in Fig. S4a†), the EDX spectra of Co-L, Ga-K and C-K clearly reveal that no coking of the Co particles during DRM operation took place. In contrast, the same treatment of Co_3_O_4_/SiO_2_ induces significant coking (Fig. 4Sc–e†). In Fig. S4c,† three areas with Co particles are marked. A clearly visible halo-like feature surrounding the Co particles is visible, which by analysis of the EDX spectra is identified as carbon encapsulating the Co particles. Additionally, extended flake-like carbon features are often observed decorating areas with Co particles (Fig. S4d and e†). Correlating the PXRD and SEM results, we infer that the Co particles on SiO_2_ give rise to deactivation by formation of graphite species arising from methane decomposition. Beneficial DRM operation using β-Ga_2_O_3_ as a support for Co particles might be traced back to the presence of CO_2_ during the dehydrogenation of light alkanes (*e.g.*, methane, ethane, propane or ethylbenzene).^57,78–80^ CO_2_ has been reported to act in a dual role: favor the dehydrogenation process, but in terms of decoking, at the same time, it facilitates the reaction by accelerating the Boudouard reaction. Specifically on β-Ga_2_O_3_, depending on the degree of reduction, a more vacancy-dominated pathway involving unsaturated Ga sites, or surface-bound heterolytic dissociation has been put forward.^56^ In the latter pathway, the alkane (*e.g.*, propane) is activated by heterolytic dissociation at Ga_2_O_3_ sites to form Ga-H and Ga-OR species, whereby the latter are decomposed to the products.^56^

### 3.3. *In situ* and *operando* powder X-ray diffraction studies of Co_3_O_4_/β-Ga_2_O_3_ during hydrogen reduction and DRM operation

Fig. 1 already revealed, that the reduction pre-treatment leads to a strong increase in DRM activity, especially in the temperature window 650 °C to 800 °C. We, thus, performed additional *in situ* and *operando* PXRD measurements to follow the structural evolution and to correlate the activity increase with potential structural transformations.

The initial state of the sample is a mixture of β-Ga_2_O_3_ and Co_3_O_4_ (Fig. 3). During the reduction step, Co_3_O_4_ transforms into α-Co with the Cu-type of crystal structure (Fig. 3a). The reduction of Co_3_O_4_ starts around 450 °C, indicated by water formation (*m*/*z* 18 and 17) and decreasing hydrogen (*m*/*z* 2) concentration, as well as by the diffuse intensity in the XRD pattern around the reflection position for Co, reaching the highest rate at around 600 °C. This effect is coupled to a broadening and intensity increase of the β-Ga_2_O_3_ reflections, which are likely due to defect formation and further crystallisation, respectively. Over time and temperature the slope of the signal for *m*/*z* 17/18 and 2 changes. This is accompanied by a reduction of the full-width-at-half-maximum of the Co reflection at 51.5°, which is shifted to lower 2*θ* due to thermal expansion. Subsequently, the water and hydrogen levels decrease further as the temperature stabilized at 800 °C (Fig. S6†). However, during further heating, the Co reflection is further shifting to lower 2*θ*. While this could be assigned to thermal expansion, this would not fit the observation under DRM conditions (see below), where the reflection is shifted to higher 2*θ* values with temperature. More likely, the shift is a combination of thermal expansion and the beginning of RMSI, leading to the incorporation of Ga into the Co lattice and, thus, the formation of a Co(Ga) alloy. Indeed, the reflection matches the one of the substitutional alloy Co80Ga20 (Fig. 3b).^73^ The appearance of the substitutional alloy at the beginning of the RMSI stage is in accordance with the Co–Ga phase diagram.^81^ With time, the composition gets more Ga-rich, as more elemental Ga is formed by reduction, until the intermetallic compound CoGa appears (Fig. 3b). A trace of Co80Ga20 is still present (at 51.1°), most likely due to diffusion limitation in larger particles.

During DRM, both Co80Ga20 and CoGa are, therefore, present at the beginning of heating (Fig. 4a). Up to 550 °C the reflections shift to lower 2*θ* values due to thermal expansion and the intensity of Co80Ga20 decreases due to the further ongoing RMSI process Following the *m*/*z* 2 and 28 profiles, indicating catalytic activity in DRM, the conversion of the CH_4_/CO_2_ mixture starts at around 450 °C, thus at a temperature when the material is mostly in the state of CoGa/β-Ga_2_O_3_. With increasing temperature, the activity for H_2_ and CO formation is increasing until the decomposition of the intermetallic compound starts (550 °C) allowing to assign the catalytic activity to CoGa/β-Ga_2_O_3_.

Above 550 °C, the CoGa reflections decrease in intensity and shift to higher 2*θ* values, while the reflections of Co80Ga20 gain intensity. This indicates a loss of Ga from CoGa by oxidation to Ga_2_O_3_ resulting in the decomposition of the intermetallic compound. Also Co80Ga20 is further oxidized until full loss of Ga, shown by the shift of the Co80Ga20 reflections to higher 2*θ* with temperature due to loss of Ga.^81^ At 800 °C, a mixture of cubic and hexagonal Co coexists on β-Ga_2_O_3_ (Fig. 4b), which is maintained upon cooling to ambient temperature. However, at room temperature hexagonal Co is the most stable allotrope, whereas cubic Co is at temperatures higher than 425 °C. While this could be explained by slow transition kinetics, stabilization by the support (*e.g.*, by epitaxy) or by stabilization of Ga in the lattice, the latter can be ruled out by Co–Ga phase diagram as the transition temperature is independent of the Ga-content of the alloy.^81^ Moreover, the loss of CoGa leads to a complete change of the selectivity pattern. The formation of CO continues, while the hydrogen formation rate decreases, which can be explained by the reverse water–gas shift activity of β-Ga_2_O_3_.^61^ Upon reaching 800 °C and during the isothermal section, the catalytic activity decreases – likely by slight sintering (Fig. S6†).

The XRD patterns during cooling down after reduction and DRM are highlighted in Fig. S7 and S8,† respectively, and revealed no structural changes.

### 3.4. Nanoscale structure of Co_3_O_4_/β-Ga_2_O_3_ after hydrogen reduction and DRM operation

**Fig. 5:**
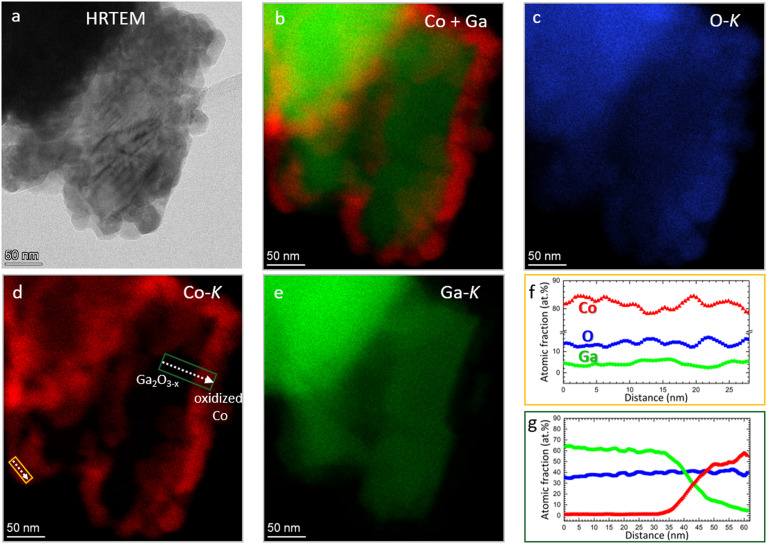
(Scanning) transmission electron microscopy analysis of as-calcined Co_3_O_4_/β-Ga_2_O_3_. Panel a: HRTEM of a β-Ga_2_O_3_ grain covered by Co_3_O_4_ particles. Panel b: Overlay of Co-K (green) and Ga-K (red) EDX mappings. Panels c–e: elemental EDX mappings of O-K (blue), Co-K (red), and Ga-K (green), respectively. Panels f and g: elemental line profiles along the orange and green-framed arrows shown in panel e, respectively. Standard deviations in panel f of O, Co, and Ga are ±2 at%, ±2 at%, and ±1 at%, respectively. Standard deviations in panel g of O, Co, and Ga are ±3 at%, ±2 at%, and ±3 at%, respectively.

Electron microscopy analysis of the as-calcined Co_3_O_4_/β-Ga_2_O_3_ sample (Fig. 5) in essence confirms the XRD patterns (*cf.*Fig. 2). The individual β-Ga_2_O_3_ grains are covered by irregular ∼50 nm-sized oxidized Co_3_O_4_ grains (panel a). The majority of Co, therefore, is oxidized (Fig. 5, panel b–g). Interestingly, all observed areas with oxidized Co are located in direct proximity to Ga-oxide with a lower oxygen content than Ga_2_O_3_ indicating the presence of a second Ga-phase with less oxygen Ga_2_O_3−*x*_, as shown on the left side in Fig. 5, panel f, where the Ga content is ∼60 at% and O ∼ 40 at%. In line with the electron microscopy results, partial formation of reduced Ga_2_O_3−*x*_ species (*e.g.*, Ga_2_O) upon thermal annealing and/or evaporation is a well-documented phenomenon.^82^

**Fig. 6:**
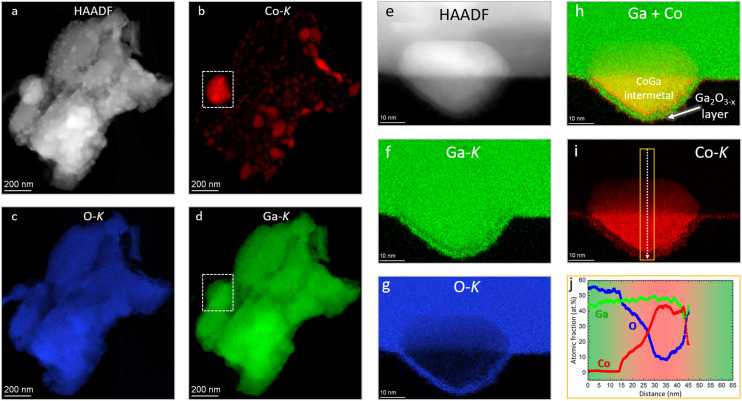
(Scanning) transmission electron microscopy analysis of the hydrogen pre-reduced Co_3_O_4_/β-Ga_2_O_3_. Panel a: mesoscale HAADF overview image. Panels b–d: elemental Co-K (red), O-K (blue) and Ga-K (green) EDX mappings. The white frame in panels b and d indicate Co–Ga intermetallic formation. Panel e: nanoscale HAADF image highlighting a single Co–Ga nanoparticle. Panels f–i: elemental Ga-K (green), O-K (blue), Co + Ga (red + green) and Co-K (red) EDX mappings, respectively. Panel j: line profiles along the arrow shown in panel i. Standard deviations in panel j of O, Co, and Ga are ±2.22 at%, ±2.15 at%, and ±3.42 at%, respectively.

**Fig. 7:**
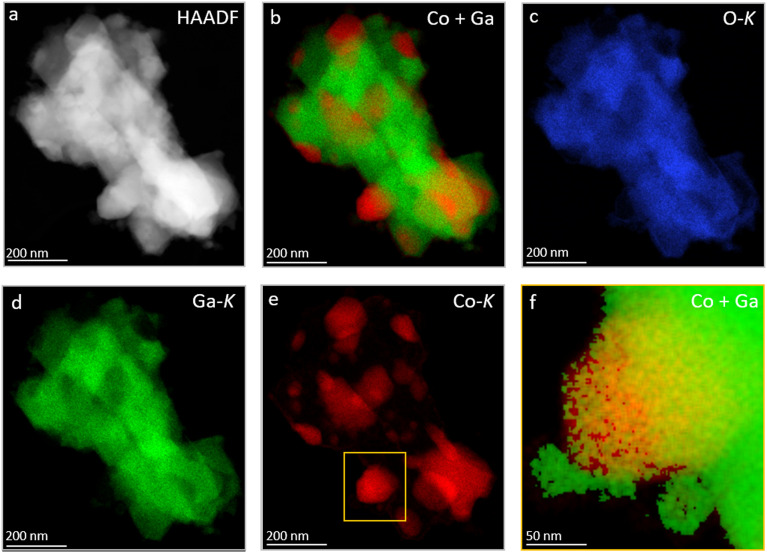
(Scanning) transmission electron microscopy analysis of the DRM-treated Co_3_O_4_/β-Ga_2_O_3_ with prior hydrogen reduction at 800 °C. Panel a: HAADF overview image highlighting metallic nanoparticles supported on Ga_2_O_3_. Panel b: Ga–Co overlay of individual elemental mappings. Panels c–e: elemental O-K (blue), Ga-K (green), and Co-K (red) EDX mappings, respectively. Panel f: zoomed-in overlay of Co and Ga signals from the yellow-framed area in panel e, showcasing a Co nanoparticle with Ga_2_O_3_ particles positioned on its surface.

After hydrogen reduction at 800 °C (Fig. 6) – corroborating the XRD results – the Co_3_O_4_ particles are reduced and bimetallic Co–Ga particles are formed, which are identified by XRD as the intermetallic compound CoGa. At the nanoscale, all individual CoGa particles are covered by an oxygen-deficient Ga_2_O_3−*x*_ layer (*cf.* line profile in Fig. 6j). Given the propensity of Ga_2_O_3_ to readily lose oxygen upon thermal annealing, we might tentatively explain the formation of this layer by formation of mobile Ga_2_O_3−*x*_ species upon hydrogen reduction and the according encapsulation of the CoGa particles by Ga_2_O_3−*x*_. Electron microscopy analysis directly confirms the reactive metal–support interaction state of Co on β-Ga_2_O_3_ inferred from the *ex situ* and *in situ* XRD experiments. After a subsequent DRM treatment up to 800 °C, the structural situation gets more complex (Fig. 7): corroborating the XRD patterns, also EDX measurements (Fig. 7, panel a, d and e) confirm that the CoGa intermetallic particles are decomposed into mostly metallic Co particles embedded in the Ga_2_O_3_ or Ga_2_O_3−*x*_ matrix and no signs of coking are detectable. Most interestingly, Fig. 7 panel f reveals on many metallic Co particles a Ga_2_O_3_ layer in close proximity. This specific Ga_2_O_3_ species is a direct result of the decomposition of the CoGa particles and creates an additional Co–Ga_2_O_3_ interface apart from the interface created by Co socketed into the main supporting Ga_2_O_3_ matrix. Note that this state is entirely different from the one that is entered by a direct DRM treatment without hydrogen pre-reduction (Fig. S9 and S10†). In that case, XRD indicates the presence of a CoGa_2_O_4_ spinel phase and EDX measurements reveal, that the Ga_2_O_3_ surface is encrusted with CoGa_2_O_4_ particles a few tens of nanometers thick. The stoichiometric evolution of Ga_2_O_3_ to CoGa_2_O_4_ is visualized by the line profile in Fig. S10,† panel f.

### 3.5. Surface chemical characterization by X-ray photoelectron spectroscopy

**Fig. 8:**
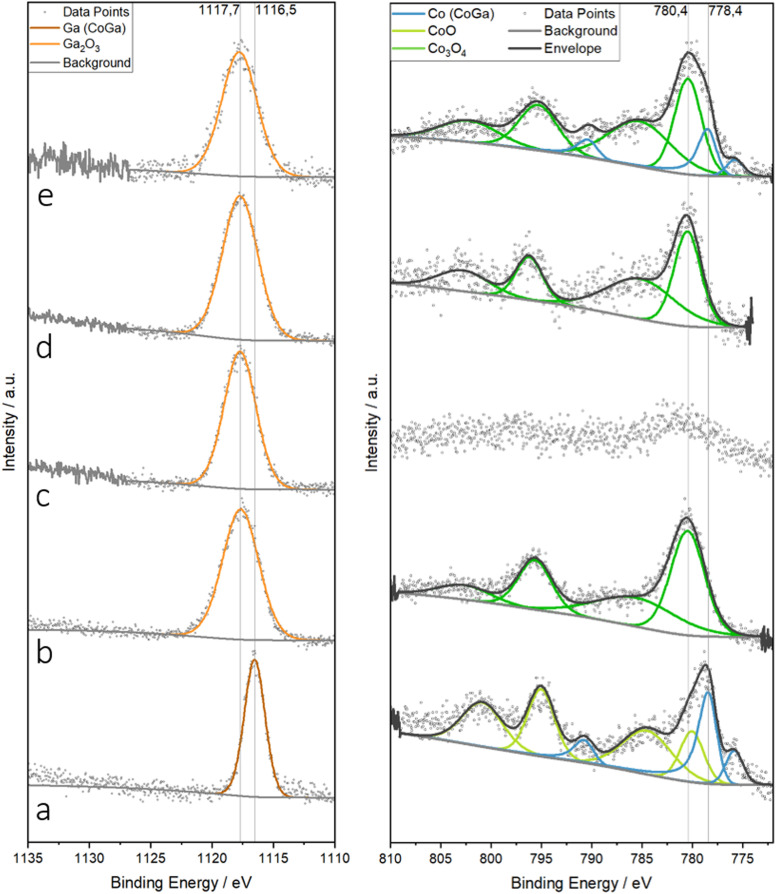
High-resolution X-ray photoelectron spectra of the Ga 2p (left) and Co 2p (right) region of Co_3_O_4_/β-Ga_2_O_3_ after selected treatments in comparison (panel b–e) in comparison with a CoGa intermetallic compound (panel a). Panel a: CoGa intermetallic compound in the as-prepared state. Panel b: Co_3_O_4_/β-Ga_2_O_3_ as calcined. Panel c: Co_3_O_4_/β-Ga_2_O_3_ after hydrogen reduction at 800 °C. Panel d: Co_3_O_4_/β-Ga_2_O_3_ after DRM. Panel e: Co_3_O_4_/β-Ga_2_O_3_ after DRM with prior hydrogen reduction at 800 °C. Binding energies of the metallic and oxidized surface states are marked by dashed lines.

In light of the substantial bulk structural and chemical changes, we monitored the according changes in the near-surface region by *ex situ* X-ray photoelectron spectroscopy (Fig. 8). We show the changes of the Ga 2p and Co 2p region as a function of treatment, referenced to the spectra of unsupported single-phase CoGa (panel a). The latter shows a single narrow Ga 2p_3/2_ signal at a binding energy of 1116.5 eV, typical of metallic Ga.^83–85^ A strong metallic component is also seen in the respective Co 2p spectrum (light green fit, main Co 2p_3/2_ at 778.4 eV).^58^ In addition, an oxidized Co 2p_3/2_ component with its satellite features at 780.4 eV is also seen, matching that of CoO.^58^ With respect to the overall shape and binding energy, all other Ga 2p spectra (panels b–e) are similar. They are dominated by a strongly oxidized Ga^3+^ component at *ca.* 1117.7 eV,^83–85^ irrespective of the treatment, in clear agreement with the electron microscopy analysis. Even after hydrogen reduction (panel c), only oxidized Ga is seen, which matches the *ca.* 5 nm thick oxygen-deficient Ga_*x*_O_*y*_ layer covering the intermetallic CoGa particles (*cf.*Fig. 6). The corresponding Co 2p spectrum (right side of panel c) reveals that Co is almost entirely shielded also due to the CoGa-covering Ga_*x*_O_*y*_ layer. The according peak data are detailed in Table S2.† In accordance with STEM-EDX (Fig. 7) and *operando* XRD (Fig. 4), the Co 2p spectrum in panel e features both a metallic and an oxidic Co component.

## 4. Conclusions

We have highlighted the potential of a Co_3_O_4_/β-Ga_2_O_3_ material as a promising DRM catalyst, rivalling known noble and/or non-noble metals-modified Co-based catalysts in terms of activity and coking resilience. In due course, we showed that while activation of Co_3_O_4_/β-Ga_2_O_3_ directly in the DRM mixture is only possible to a limited extent due to the formation of a surface-bound CoGa_2_O_4_ spinel phase, hydrogen pre-reduction yields reduction of the initial Co_3_O_4_ particles on β-Ga_2_O_3_ to α-Co metal and further to CoGa on β-Ga_2_O_3_. Hence, Co is able to enter a reactive metal–support interaction stage (RMSI) on β-Ga_2_O_3_. This also leads to coverage of the individual CoGa particles by a strongly oxygen-deficient Ga_*x*_O_*y*_ layer, arising from the transport of mobile Ga–O species onto the intermetallic particles. Oxygen-deficient Ga sites are already present in the as-calcined state, but are more abound after hydrogen reduction, as evidenced by STEM/EDX. Intermetallic compound formation is reversed by subsequent treatment in a DRM mixture, causing decomposition of CoGa into cubic α-Co particles on β-Ga_2_O_3_, *i.e.*, after CoGa decomposition, the formed Co–Ga_2_O_3_ interface remains exposed and accessible to reactants, based on electron microscopy data. These two phases do not form the slightly active CoGa_2_O_4_ spinel, thus leading to excellent activity of the material. The decomposition temperature of CoGa can be directly related to the strong increase in DRM activity by *operando* XRD measurements. In comparison to SiO_2_, β-Ga_2_O_3_ shows a better overall DRM activity with respect to coking resilience within the limitation of comparing activity without actual TOF values, which are inaccessible due to the *in situ* formation of the active sites.

These studies in turn pave the way to the potential redox activation of isolated Co–Ga intermetallic compounds. Similar to the Cu–In system, due the selective oxidation of Ga, we expect the oxidative decomposition of Co–Ga intermetallic compounds with varying stoichiometry, *e.g.*, CoGa or CoGa_3_, and a potentially improved DRM performance due to beneficial extents of Co–Ga_2_O_3_ phase boundary dimensions and the accordingly higher activity, stability and anti-coking behavior.

## Conflicts of interest

There are no conflicts to declare.

## Supplementary Material

CY-015-D5CY00179J-s001

## Data Availability

Data for this article are available at Researchdata at https://doi.org/10.48323/fv702-2t003.
