# Supplementary material for: The role of Co–Ga2O3 interfaces in methane dry reforming
Source: Catal Sci Technol. 2025 May 7;15(12):3667–80. doi: 10.1039/d5cy00179j (PMC12056703; doi:10.1039/d5cy00179j)
Supplement: CY-015-D5CY00179J-s001 [file CY-015-D5CY00179J-s001.pdf]

Supplementary Information (SI) for Catalysis Science & Technology.
This journal is © The Royal Society of Chemistry 2025

Supporting Information

The role of Co-Ga_2O_3 interfaces in methane dry reforming

Thomas F. Winterstein,[1] Christoph Malleier,[1] Asghar Mohammadi,[1] Roham Talei,[2]

Guido Schmitz,[2] Nicolas Bonmassar,[2] Jesus Andrade,[3] Marc Armbrüster,[3,4] Simon Penner[1]

[1]*Institute of Physical Chemistry, University of Innsbruck, Innrain 52c, A-6020 Innsbruck*

[2]*Institute for Materials Science, University of Stuttgart, Heisenbergstr. 3, 70569 Stuttgart, Germany*

[3]*Materials for Innovative Energy Concepts, Chemnitz University of Technology, Straße der Nationen 62, 09111 Chemnitz, Germany*

[4]*Solid State and Material Chemistry, Technical University Darmstadt, Peter-Grünberg Str. 12, 64287 Darmstadt*

Corresponding author: S. Penner, simon.penner@uibk.ac.at, +4351250758003

Keywords: CoGa, intermetallic compound, activation, dry reforming, reactive metal-support interaction

Section A DRM profile of Co_3O_4/β-Ga_2O_3 without hydrogen pre-treatment

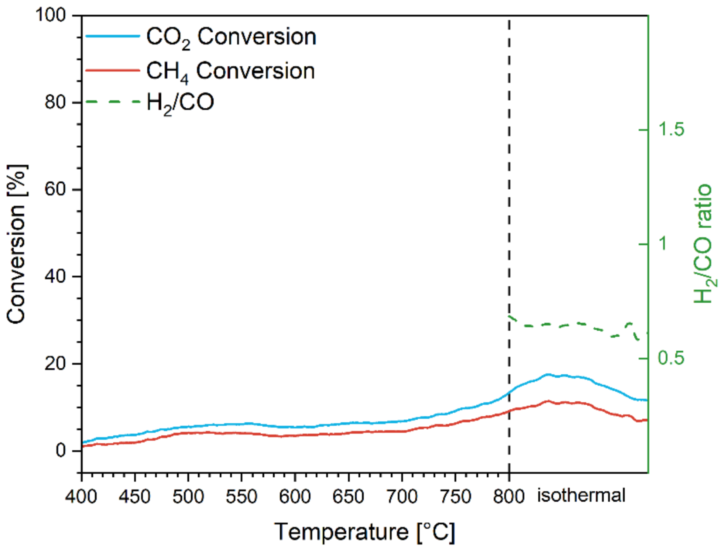

Figure S1: DRM profile of Co_3O_4/β-Ga_2O_3 without hydrogen pre-treatment. Experimental conditions as in Figure 1.

Section B Rietveld data of the ex situ collected PXRD patterns

Table S1: Rietveld data of the ex situ collected PXRD patterns

System	Phase	Weight Fraction [wt.-%]
as calcined	Co_3O_4	14.6
	Ga_2O_3	85.4
after DRM	$CoGa_2O_4$	42.4
	Ga_2O_3	57.6
after H_2	CoGa	23.2
	Ga_2O_3	76.8
after H_2+DRM	α-Co<br>ε-Co	5.4<br><0.2 below limit of quantification
	Ga_2O_3	94.5

Section C Reference catalytic measurements of blank reactor, SiO_2, β-Ga_2O_3, Co_3O_4, and $CoGa_2O_4$

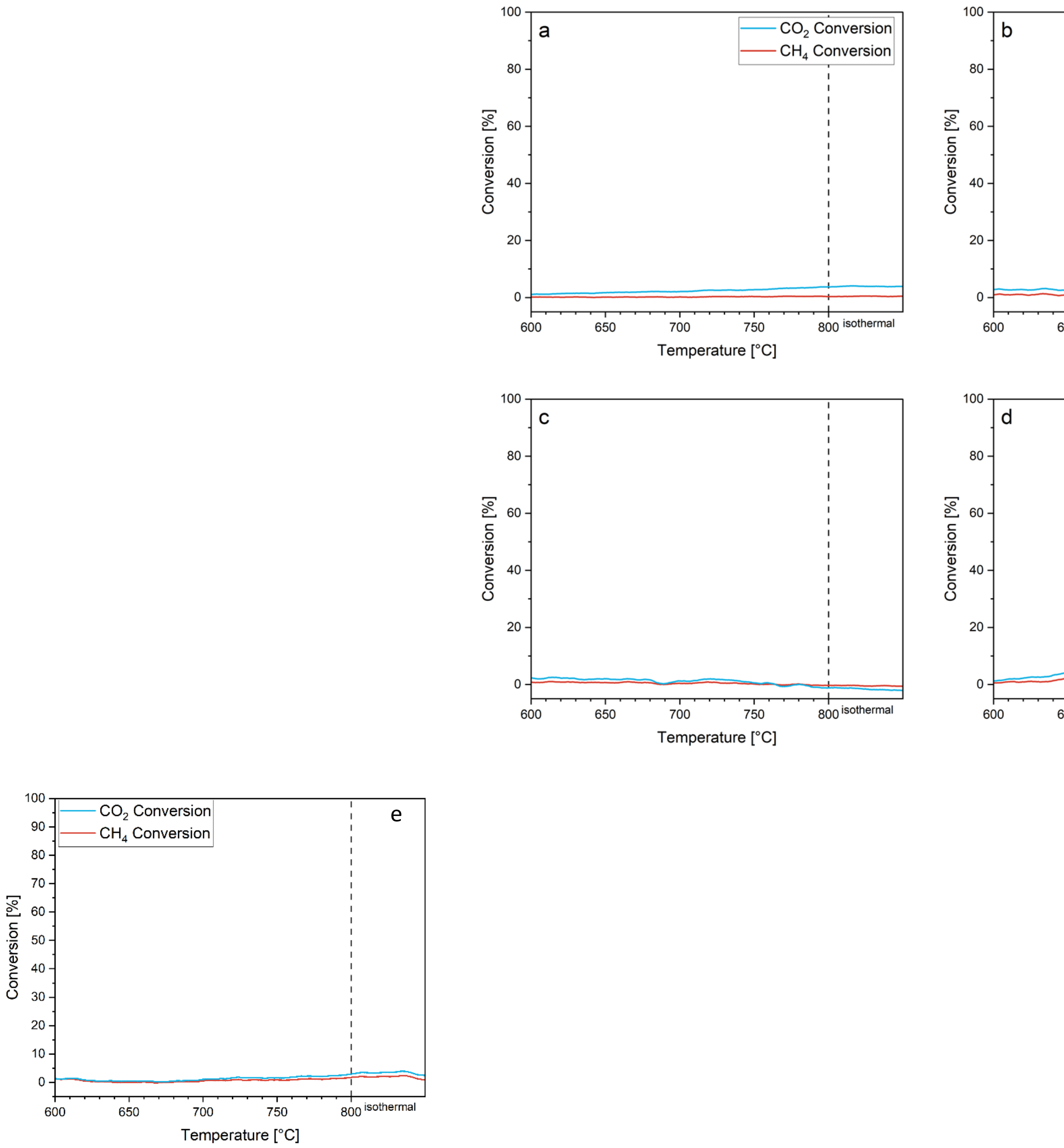

Figure S2: DRM profiles of β-Ga_2O_3 (Panel a), SiO_2 (Panel b), blank reactor (Panel c), Co_3O_4 (Panel d) and $CoGa_2O_4$

Section D Details of the Co/SiO_2 catalyst

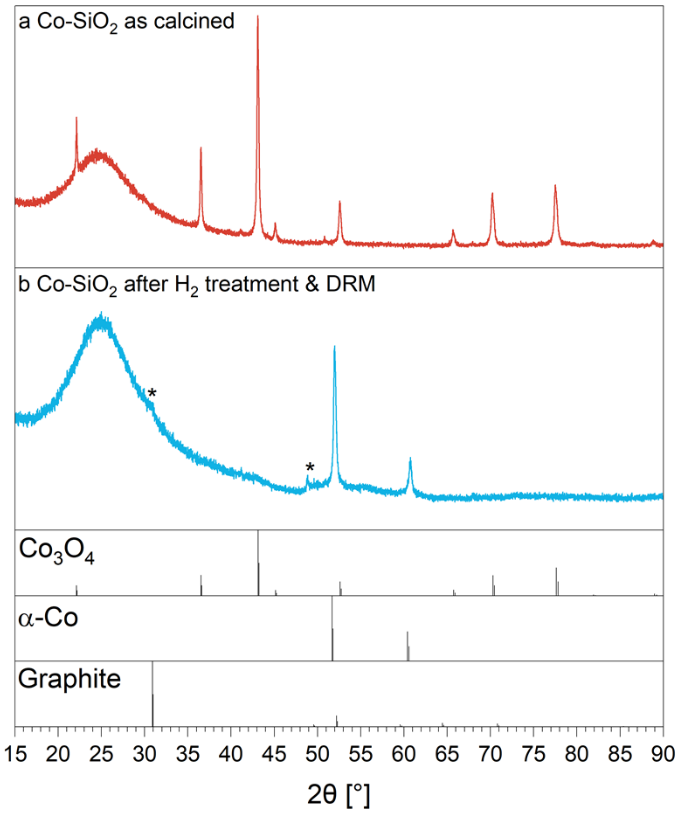

Figure S3: XRD patterns of Co_3O_4/SiO_2 in Panel **a**: as-calcined state, Panel **b**: after DRM operation at 800 °C with prior hydrogen reduction at 450 °C. The asteriks marks the respective reflections of graphite. Structure ICSD-Numbers & references: graphite 76767 [1], Co_3O_4 36256 [62], α-Co 136039 [70].

[1] P. Trucano, R. Chen, Structure of graphite by neutron diffraction, *Nature* 1975, **258**, 136-137

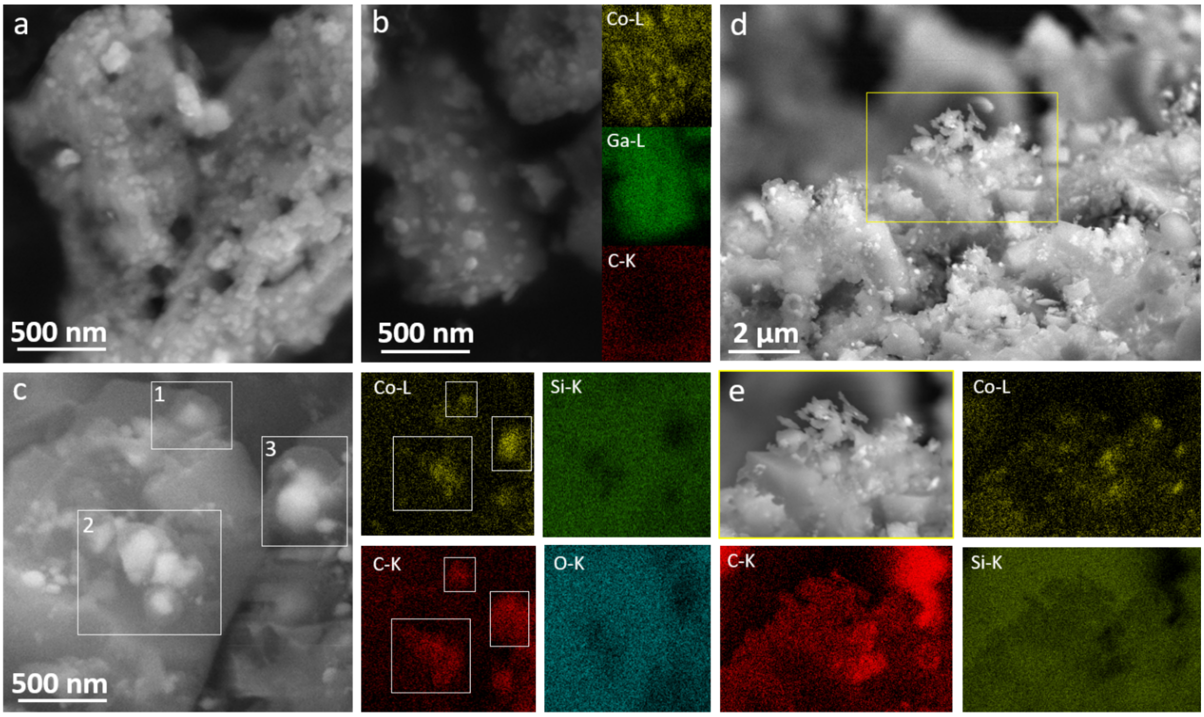

Figure S4: SEM analysis of Co_3O_4/β-Ga_2O_3 and the Co_3O_4/SiO_2 reference powder catalyst in the as-calcined state and after a hydrogen pre-reduction (1 bar flowing H_2, 550 °C, 1 h for Co_3O_4/β-Ga_2O_3; 1 bar flowing H_2, 450 °C, 10 min for Co_3O_4/ SiO_2) – DRM cycle (conditions as in Figure 1). Panel **a**: Co_3O_4/β-Ga_2O_3 as-calcined. Panel **b**: Co_3O_4/β-Ga_2O_3 after reduction at 550 °C/ DRM operation. Panel **c**: Co_3O_4/SiO_2 after reduction at 450 °C/ DRM operation. Panel **d** and **e**: Detail of the catalyst shown in Panel **c**. Panels **b**, **c** and **e** exhibit EDX maps of the Co-L, Ga-L, Si-K, C-K and O-K edges. The insets in Panel **c** (marked 1,2 and 3) are added as a guide to the eye to pinpoint the exact same locations in the EDX maps. Panel **e** is a magnification of the yellow rectangle shown in Panel **d**.

Section E: XRD pattern of the as-synthesized untreated CoGa intermetallic compound

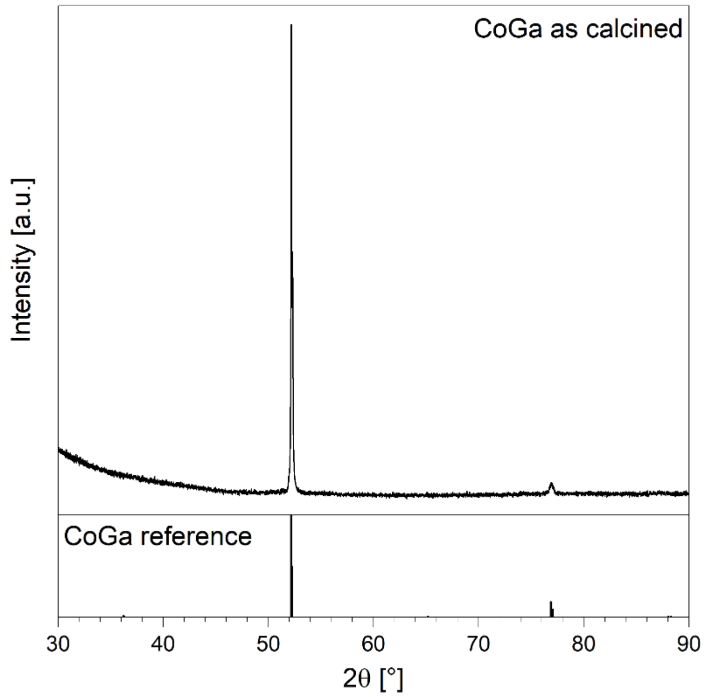

Figure S5: XRD pattern of the as-synthesized untreated CoGa intermetallic compound. Structure ICSD-Number & reference: CoGa 657494 [67].

Section F Isothermal mass spectra during reduction and DRM conditions at 800 °C

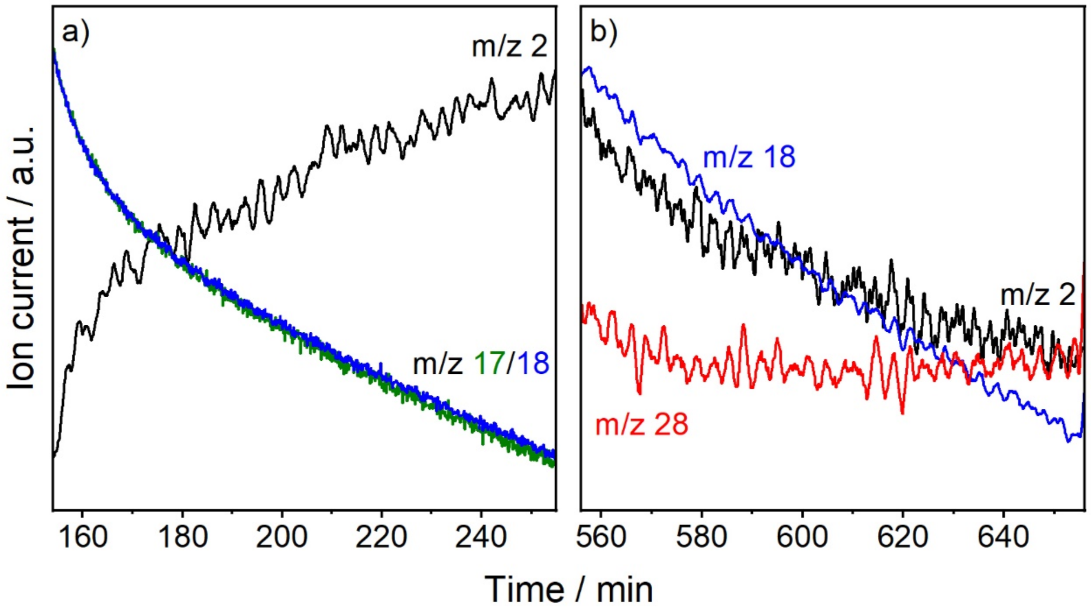

Figure S6: Isothermal mass spectra during (a) reduction and (b) DRM conditions, both at 800°C.

Section G XRD Characterization after re-cooling

The XRD patterns during cooling down after reduction and DRM can be observed on Figure S7 and S8, respectively. From both figures no new structural change can be observed, besides what was pointed out previously. In both figures, the reflections underwent to thermal shrinking during the cooling down part.

Regarding Figure S7, the reflection of CoGa starting at 51.6° 2θ should increase to very high intensities (as can be observed in Figure 3b). However, it can be observed a 'dark zone' in the regions above 51.8° which it is attributed to the misalignment of the height of the sample and the detector due to thermal expansion during reduction. This effect 'hides' the reflection corresponding to CoGa and the intensity increase related to it, which is observed in Figure 3b by comparing both plots at 800 and 50° C. This effect can also be observed in Figure 3a (first part of the reduction), but is not observable during the DRM step (Figure 4a and Figure S8) since a second sample alignment was done to correct these misalignment effects just before the DRM step (at 50 °C).

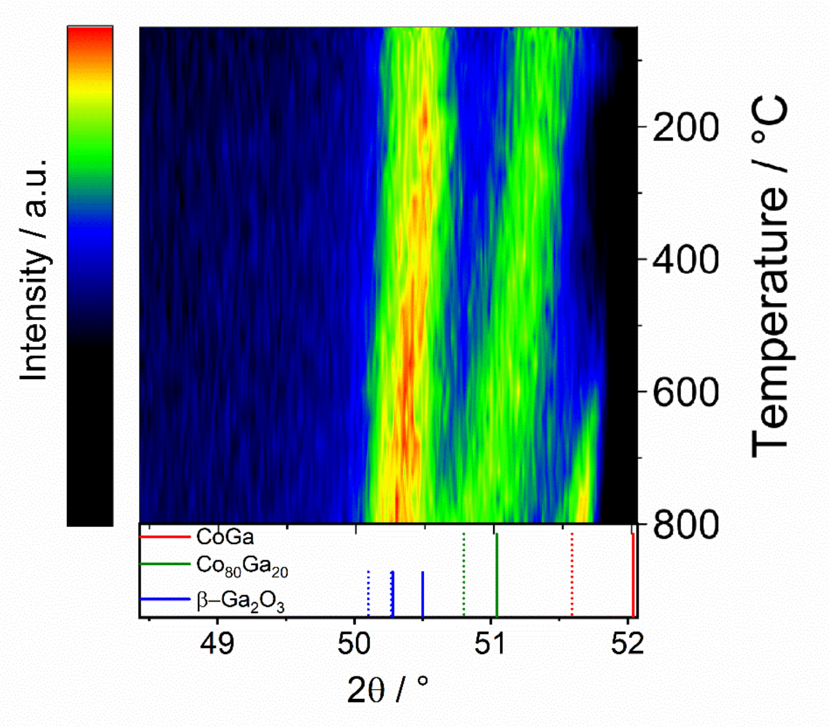

Figure S7: In situ XRD patterns collected on Co_3O_4/β-Ga_2O_3 during reduction in hydrogen while cooling down from 800 to 50 °C. The dotted lines in the inset are the calculated XRD patterns of the modified lattice parameters due to thermal expansion. [Refences from the XRD patterns are the same as the main publication].

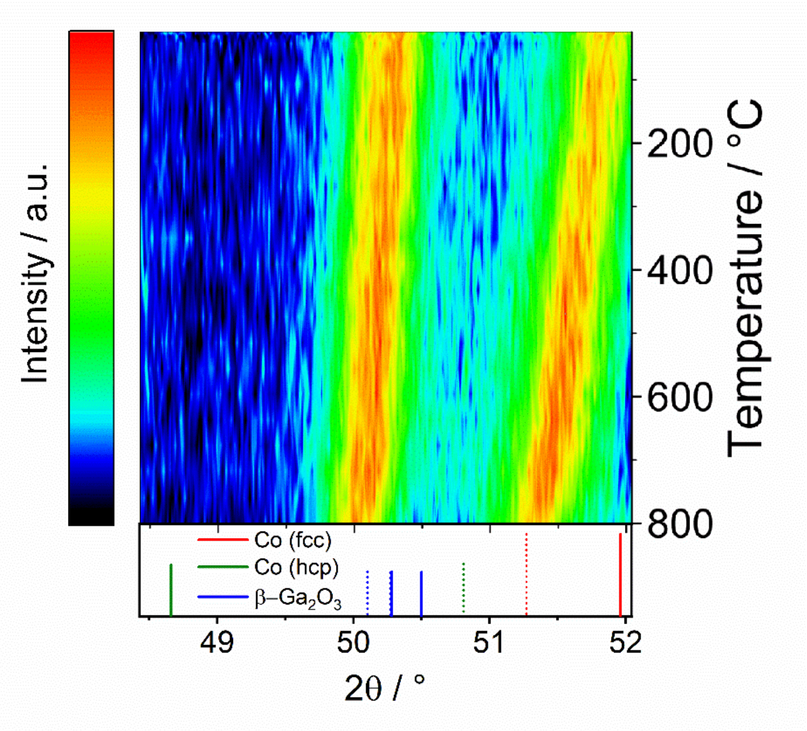

Figure S8: In situ XRD patterns collected on Co_3O_4/β-Ga_2O_3 during cooling down from 800 to 50 °C after DRM. The dotted lines in the inset are the calculated XRD patterns of the modified lattice parameters due to thermal expansion. [References from the XRD patterns are the same as the main publication].

Section H Electron microscopy analysis of the Co_3O_4/β-Ga_2O_3 catalyst after a direct DRM treatment without hydrogen pre-reduction

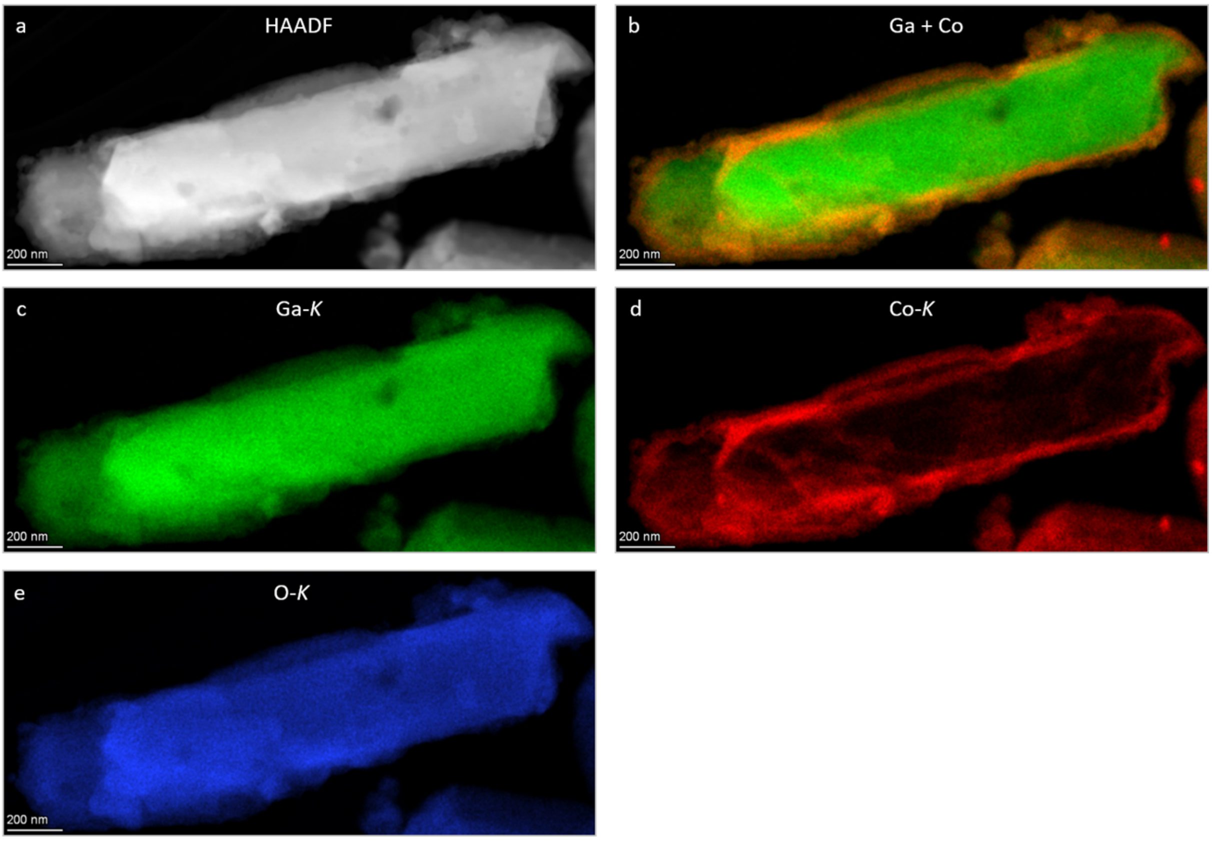

Figure S9: Mesoscale electron microscopy analysis of Co_3O_4/β-Ga_2O_3 catalyst after a direct DRM treatment with out hydrogen pre-reduction. Panel a: HAADF. Panel b: Overlay of the Ga-K and Co-K EDX intensities. Panels c-d: Individual Ga-K (c), Co-K (d) and O-K (e) EDX intensities.

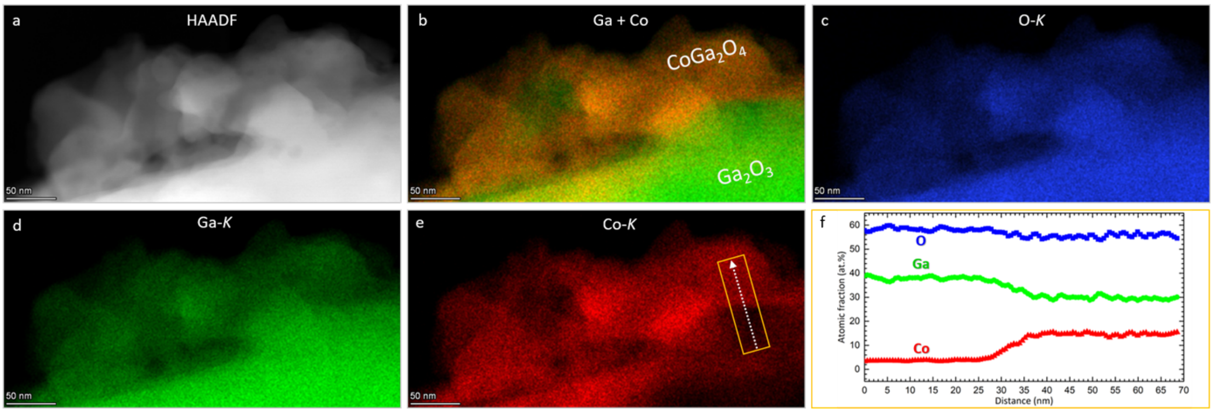

Figure S10: Nanoscale electron microscopy analysis of Co_3O_4/β-Ga_2O_3 after a direct DRM treatment without hydrogen pre-reduction. Panel a: HAADF. Panel b: Overlay of the Ga-K and Co-K EDX intensities. Panels c-d: Individual O-K (c), Ga-K (d) and Co-K (e) EDX intensities. Panel f: Line profile along the arrow shown in Panel e.

Section I Peak data of the XPS analysis

Table S2: Peak data of the Co 2p and Ga 3d XPS analysis including binding energy/ eV, full-width-at-half-maximum (FWHM), peak area/ % and peak ratio.

		Comp.	Pos. / eV	FWHM	% Area / -	Ratio / -
Co 2p	H_2 + DRM	Co 2p 3/2	780.38	3.87	31.93	1.00
		Co 2p 3/2 sat.	785.20	10.34	39.92	1.25
		Co 2p 1/2	795.98	4.46	17.45	0.55
		Co 2p 1/2 sat.	802.60	6.83	10.70	0.34
	DRM	Co 2p 3/2	780.48	3.14	33.72	1.00
		Co 2p 3/2 sat.	785.14	8.09	36.90	1.09
		Co 2p 1/2	796.21	3.10	15.25	0.45
		Co 2p 1/2 sat.	802.79	5.93	14.14	0.42
	H_2	-	-	-	-	-
		-	-	-	-	-
		-	-	-	-	-
		-	-	-	-	-
	As Calcined	Co 2p 3/2	780.45	3.95	42.33	1.00
		Co 2p 3/2 sat.	785.62	9.63	28.62	0.68
		Co 2p 1/2	795.63	3.86	19.71	0.47
		Co 2p 1/2 sat.	802.71	6.19	9.34	0.22
	Intermetallic	Co met. LMM Auger	775.82	2.04	6.53	0.52
		Co met. 3/2	778.44	2.00	16.12	1.28
		Co met. 1/2	790.75	2.20	4.42	0.35
		Co 2p 3/2	780.02	2.77	12.57	1.00
		Co 2p 3/2 sat.	784.50	5.83	22.76	1.81
		Co 2p 1/2	795.02	3.14	18.91	1.50
		Co 2p 1/2 sat.	800.88	4.72	18.68	1.49

		Comp.	Pos. / eV	FWHM	% Area / -	Ratio / -
Ga 3d	H_2 + DRM	Ga^{3+} 3d 5/2	1117.77	3.42	100.00	1.00
	DRM	Ga^{3+} 3d 5/2	1117.72	3.40	100.00	1.00
	H_2	Ga^{3+} 3d 5/2	1117.72	3.07	100.00	1.00
